# Uncovering the Ancestry of B Chromosomes in *Moenkhausia sanctaefilomenae* (Teleostei, Characidae)

**DOI:** 10.1371/journal.pone.0150573

**Published:** 2016-03-02

**Authors:** Ricardo Utsunomia, Duílio Mazzoni Zerbinato de Andrade Silva, Francisco J. Ruiz-Ruano, Cristian Araya-Jaime, José Carlos Pansonato-Alves, Priscilla Cardim Scacchetti, Diogo Teruo Hashimoto, Claudio Oliveira, Vladmir A. Trifonov, Fábio Porto-Foresti, Juan Pedro M. Camacho, Fausto Foresti

**Affiliations:** 1 Departamento de Morfologia, Instituto de Biociências de Botucatu, Universidade Estadual Paulista, Botucatu, São Paulo, Brazil; 2 Departamento de Genética, Universidad de Granada, Granada, Spain; 3 CAUNESP, Universidade Estadual Paulista, Jaboticabal, São Paulo, Brazil; 4 Institute of Molecular and Cellular Biology SB RAS, Nobosibirsk, Russia; 5 Departamento de Ciências Biológicas, Faculdade de Ciências, Universidade Estadual Paulista, Bauru, São Paulo, Brazil; Leibniz-Institute of Plant Genetics and Crop Plant Research (IPK), GERMANY

## Abstract

B chromosomes constitute a heterogeneous mixture of genomic parasites that are sometimes derived intraspecifically from the standard genome of the host species, but result from interspecific hybridization in other cases. The mode of origin determines the DNA content, with the B chromosomes showing high similarity with the A genome in the first case, but presenting higher similarity with a different species in the second. The characid fish *Moenkhausia sanctaefilomenae* harbours highly invasive B chromosomes, which are present in all populations analyzed to date in the Parana and Tietê rivers. To investigate the origin of these B chromosomes, we analyzed two natural populations: one carrying B chromosomes and the other lacking them, using a combination of molecular cytogenetic techniques, nucleotide sequence analysis and high-throughput sequencing (Illumina HiSeq2000). Our results showed that i) B chromosomes have not yet reached the Paranapanema River basin; ii) B chromosomes are mitotically unstable; iii) there are two types of B chromosomes, the most frequent of which is lightly C-banded (similar to euchromatin in A chromosomes) (B_1_), while the other is darkly C-banded (heterochromatin-like) (B_2_); iv) the two B types contain the same tandem repeat DNA sequences (18S ribosomal DNA, H3 histone genes, MS3 and MS7 satellite DNA), with a higher content of 18S rDNA in the heterochromatic variant; v) all of these repetitive DNAs are present together only in the paracentromeric region of autosome pair no. 6, suggesting that the B chromosomes are derived from this A chromosome; vi) the two B chromosome variants show MS3 sequences that are highly divergent from each other and from the 0B genome, although the B_2_-derived sequences exhibit higher similarity with the 0B genome (this suggests an independent origin of the two B variants, with the less frequent, B_2_ type presumably being younger); and vii) the dN/dS ratio for the H3.2 histone gene is almost 4–6 times higher for B chromosomes than for A chromosome sequences, suggesting that purifying selection is relaxed for the DNA sequences located on the B chromosomes, presumably because they are mostly inactive.

## Introduction

B chromosomes are dispensable genomic elements that are present in approximately 15% of eukaryotes. These chromosomes exhibit a parasitic nature, and their interaction with the host genome determines their population frequency, which is highly dynamic as a consequence of “the arms race” between A and B chromosomes [1–3]. Because B chromosomes do not always occur in pairs, their segregation does not conform to a Mendelian system, which may facilitate transmission rates higher than 0.5 among these chromosomes, resulting in transmission advantages collectively referred to as “drive” [3, 4].

The origin of B chromosomes has been investigated in various organisms. Basically, they may arise from the A chromosomes of the current host species (intraspecific origin) or be derived interspecifically through hybridization [3]. An intraspecific origin of B chromosomes has been demonstrated, for instance, in maize [5, 6], the migratory locust [7], rye [8], and the fish *Astyanax paranae* [9]. However, examples of B chromosomes arising through interspecific hybridization have been reported in the plant genus *Coix* [10], the fish *Poecilia formosa* [11], and the wasp *Nasonia vitripennis* [12, 13].

Although the sequence composition of B chromosomes is unknown in most cases, several studies using fluorescent in situ hybridization (FISH) and next-generation sequencing (NGS) have allowed better characterization of repetitive DNA sequences and single-copy genes located on B chromosomes [14, 15]. Notably, these data provided new insights about the origin of B chromosomes [8, 16–18] and have suggested that some of the DNA contained in B chromosomes is potentially functional, as ribosomal DNA within the B chromosome of the grasshopper *Eyprepocnemis plorans* is able to yield the corresponding phenotype (i.e., a nucleolus) [19].

*Moenkhausia* (Teleostei, Characidae) is a small freshwater characid fish genus that is widely distributed in South American river basins and comprises approximately 86 species [20]. *M*. *sanctaefilomenae* shows a conserved karyotype with respect to the standard genome (2n = 50 biarmed chromosomes) but also carries micro-B chromosomes in all populations hitherto analyzed [21–25]. Specifically, several populations collected in the Tietê River basin show euchromatic, or partially or totally heterochromatic B chromosomes [21, 23–25], whereas one population collected in the Paraná River shows only the euchromatic variant, which is restricted to males [22].

Recently, after karyotyping three populations from the Tietê River basin and performing chromosome painting using a B-specific probe, Scudeler et al. [25] concluded that the heterochromatic B type had an intraspecific origin, due to sharing DNA sequences with several A chromosomes, and that it arose independently from the euchromatic B chromosome, as a painting probe produced from the former B type did not paint the latter variant. In an attempt to extend these conclusions further, in the present study, we analyzed a natural population carrying the euchromatic and heterochromatic variants by means of a combination of cytogenetic (C-banding, microdissection, chromosome painting, FISH mapping, silver staining), molecular (PCR amplification, cloning, Sanger DNA sequencing and Illumina sequencing), phylogenetic, and bioinformatics techniques, additionally providing the first report for a B-lacking population in this species.

## Materials and Methods

### Ethics Statement

Sampling was carried out on private lands, and the owners gave permission to conduct this study. The animals were captured using nets, transported to the laboratory and kept in a fish tank for 2 days, and they were anesthetized before the analyses. The animals were collected in accordance with Brazilian environmental protection legislation (Collection Permission MMA/IBAMA/SISBIO—number 3245) and the procedures for the sampling, maintenance and analysis of the fishes were performed in compliance with the Brazilian College of Animal Experimentation (COBEA) and approved (protocol 504) by the BIOSCIENCE INSTITUTE/UNESP ETHICS COMMITTEE ON THE USE OF ANIMALS (CEUA).

### Sampling, chromosome banding and DNA extraction

Individuals of *M*. *sanctaefilomenae* were sampled in 2 rivers of the Paraná River system, with 23 specimens (11 females and 12 males) being collected from the Batalha River (BR), belonging to the Tietê River basin (Bauru, SP; 22°24’23.65’S, 49°05’51.38”W), and 16 individuals (11 females and 5 males) from the Novo River (NR), in the Paranapanema River basin (Ocauçu, SP; 22°28’13.32”S, 49°55’26.17”W). These rivers are separated from each other by approximately 170 Km as the crow flies and by several hundred kilometres through the rivers connecting them. After analysis, all specimens were deposited in the fish collection of the Laboratório de Biologia e Genética de Peixes (LBP) at UNESP, Botucatu, São Paulo, Brazil, under voucher numbers LBP19830 (Batalha River) and LBP19831 (Novo River). After capture, the animals were taken to the Laboratory, transferred to an aerated 40L glass aquarium (60 x 30 x 30 cm) at 25°C and kept there until sacrifice for three days at most. Food administration was not carried out.

Before analysis, the animals were sacrificed by overdose of anaesthetic in 1% benzocaine in water. Mitotic chromosomes were obtained from cell suspensions from the anterior kidney according to Foresti et al. [26]. C-banding was carried out according to Sumner [27], and active nucleolar organizer regions (NORs) were revealed according to Howell and Black [28]. The chromosomes were classified as metacentric (m), submetacentric (sm), subtelocentric (st) and acrocentric (a) according to Levan et al. [29]. Genomic DNA (gDNA) was obtained from liver cells using the Promega Wizard Genomic DNA Purification Kit according to the manufacturer’s instructions.

### Chromosome microdissection

Cell suspensions were dropped onto clean coverslips. The coverslips were then washed in a 1x PBS solution for 1 minute, incubated in a trypsin solution (1% Trypsin, 1x PBS) for 20 seconds and washed again in 1x PBS. Finally, the preparations were stained with 5% Giemsa in PBS for 5 minutes. For the microdissection of B_1_, we used cell suspensions from individual 69693, which presented only this variant (Table 1). To microdissect the B_2_ variant, C-banding was performed to allow better identification of the heterochromatic B chromosome in the metaphase spread. In addition, one autosome from a B-carrying individual was also microdissected (pair No. 1, henceforth referred to as A_1_). It must be noted that all microdissection experiments were carried out using single-copy chromosomes.

**Table 1:** Intra- and interindividual variation in the number of B_1_ and B_2_ chromosomes in the BR population.

Id no.	Sex	Cells with 0–6 B_1_ chromosomes										Cells with 0–2 B_2_ chromosomes						Total
		0	1	2	3	4	5	6	Mean	Median	MI	0	1	2	Mean	Median	MI	
69616	M	2	2	12	3	2			2.05	2	0.31	21			0	0		21
69618	M	0	1	1	5	7			3.29	3.5	0.20	14			0	0		14
69626	F	3	13	13	2				1.45	1	0.65	31			0	0		31
69628	F	3	14	8	2	3			1.60	1	0.80	0	12	18	1.60	2	0.20	30
69676	M	0	4	21	13				2.24	2	0.22	38			0	0		38
69678	F	8	15	6					0.93	1	0.48	2	16	11	1.31	1	0.45	29
69679	M	4	0	8	18	2			2.44	3	0.23	3	22	7	1.13	1	0.31	32
69680	F	2	4	14	4				1.83	2	0.25	24			0	0		24
69682	M	4	14	4					1.00	1	0.36	22			0	0		22
69637	M	2	3	10	10				2.12	2	0.34	25			0	0		25
69693	F	0	15	14	3				1.63	2	0.28	32			0	0		32
69695	M	3	20	5					1.07	1	0.29	28			0	0		28
69696	F	0	4	24	5				2.03	2	0.14	33			0	0		33
69710	F	0	2	8	18	2			2.67	3	0.16	30			0	0		30
69713	M	1	5	13	9				2.07	2	0.29	28			0	0		28
69714	F	3	26	4					1.03	1	0.21	33			0	0		33
69729	M	0	6	9	8	6	2	1	2.75	3	0.35	32			0	0		32
69730	F	0	0	4	11	11			3.27	3	0.19	26			0	0		26
69731	M	11	18	10					0.97	1	0.54	39			0	0		39
69739	M	0	13	16	2				1.65	2	0.24	0	29	2	1.06	1	0.06	31
69740	M	0	5	4	9				2.22	2.5	0.31	18			0	0		18
69741	F	1	5	10	12				2.18	2	0.34	28			0	0		28
69743	F	0	1	10	20	3			2.74	3	0.15	34			0	0		34
**Mean**									1.97	2	0.32				0.22	0.22	0.26	
**SE**									0.15	0.17	0.03				0.25	0.26	0.08	

MI = mitotic instability index, F = female, M = male, SE = standard error.

Each microdissected chromosome was transferred to a micropipette containing a collection solution (1.5 μg/μl proteinase K, 0.1% SDS, 0.1% Triton X-100, 1 mM EDTA, 10 mM Tris-HCl, pH 8.0, 10 mM NaCl) and placed for 1 h at 60°C in a moist chamber. Then, the pipette tips were broken into a 0.2 ml microtube containing 5 μl of sterile MilliQ water, followed by amplification using the GenomePlex Single Cell Whole Genome Amplification Kit (WGA-Sigma).

### Whole-genome sequencing (WGS) and satellite DNA identification

To perform a deeper search for satDNAs in the *M*. *sanctaefilomenae* genome, we sequenced gDNA from two individuals collected from the Batalha River (carrying up to 6B) and the Novo River (0B) on the Illumina HiSeq2000 platform, yielding 2x101 bp paired-end reads. After a quality trimming step (filtering out reads with less than 90% of bases showing a quality lower than Q20) with Trimmomatic [30], we sampled 200,000 pairs of reads (100,000 reads from each population) for clustering using RepeatExplorer [31] considering paired-end reads, with clustering and assembly overlap lengths equal to 55 and 40 bp, respectively. For this analysis, we also built a custom database of repeated sequences by running RepeatModeler [32] on the assembled *A*. *mexicanus* genome (GenBank accession number APWO00000000.1) as a complement to RepBase for cluster annotation. This custom database resulted in 1,243 sequences, consisting of 589,736 bp and N50 = 613 bp. We subsequently searched for clusters of satDNA families (i.e., unannotated) with sphere or ring shapes and a graph density higher than 0.1. Next, we manually processed the assembled contigs with Geneious Pro v8.04 using the High Sensitivity/Slow option to visualize dotplot graphics to detect tandem repetitions. We then split these sequences into monomers, aligned them and obtained a consensus sequence of the monomeric units.

### Repetitive DNA probes

5S rDNA, U2 snDNA and H3 histone gene probes were obtained via PCR directly from the genome of *M*. *sanctaefilomenae* using both primers described previously [6, 33–35] and others described here, as follows. To yield an optimum-sized 18S rDNA probe (≈600 bp), a new set of primers was designed based on sequences of the organisms available in GenBank: 18S6F (5’-CTCTTTCGAGGCCCTGTAAT-3’) and 18S6R (5’-CAGCTTTGCAACCATACTCC-3’). Probes of for two satDNAs were obtained using the following divergent primers: MS3F (5’-TGGTTCCCAATTTGCAATCAAG-3’) and MS3R (5’-ATCGGACCTTTCTTCGCTTTACA-3’); MS7F (5’-CACAAGCCTTATGTTCACCATGA-3’) and MS7R (5’-GTACAGTAAAGTTGTAAGTGGT-3’).

### FISH

Prior to the FISH experiments, all probes were labelled with digoxigenin-11-dUTP or biotin-16-dUTP. The painting probes were labelled using the GenomePlex (WGA3 Reamplification Kit-Sigma) following the manufacturer’s protocol, and the repetitive DNA probes were labelled via PCR.

FISH was performed under high stringency conditions using the method described by Pinkel et al. [36]. The pre-hybridization conditions were different according to the probes used. Thus, slides probed with repetitive sequences were incubated with RNAse (50 μg/ml) for 1 h at 37°C, and the chromosomal DNA was denatured in 70% formamide/2x SSC for 5 min at 70°C. For each slide, 30 μl of hybridization solution (containing 200 ng of each labelled probe, 50% formamide, 2x SSC and 10% dextran sulphate) was denatured for 10 minutes at 95°C, then dropped onto the slides and allowed to hybridize overnight at 37°C in a moist chamber containing 2x SSC. Slides probed with whole-chromosome paints were incubated with 0,005% pepsin/10 mM HCl for 10 min, and the chromosomal DNA was denatured in 70% formamide/2x SSC for 3 min at 70°C. For each slide, 30 μl of hybridization solution (containing 200 ng of labelled probe, 50% formamide, 2x SSC, 10% dextran sulphate and 3 μg of salmon sperm DNA) was denatured for 10 minutes at 85°C and allowed to pre-hybridize for 30 min at 37°C, then dropped onto the slides, followed by sealing with rubber cement and hybridization at 37°C in a moist chamber containing 2x SSC for 36 h. Post-hybridization, all slides were washed in 0.2x SSC/15% formamide for 20 min at 42°C, followed by a second wash in 0.1x SSC for 15 min at 60°C and a final wash at room temperature in 4x SSC, 0.5% Tween for 10 min. Probe detection was carried out with avidin-FITC (Sigma) or anti-digoxigenin-rhodamine (Roche), and the chromosomes were counterstained with DAPI (4’,6-diamidino-2-phenylindole, Vector Laboratories) and analyzed using an optical photomicroscope (Olympus BX61). Images were captured using Image Pro plus 6.0 software (Media Cybernetics). From each individual, a minimum of 10 cells was analyzed to confirm the FISH results and estimate the number of B chromosomes per cell.

### DNA amplification, cloning and sequencing

We amplified, cloned and sequenced partial H3 histone genes and both satDNAs from various samples, including a microdissected euchromatic B chromosome (B_1_), a microdissected heterochromatic B chromosome (B_2_), gDNA 0B from *M*. *sanctaefilomenae* from the Novo River (0B gDNA) and 0B gDNA from *A*. *fasciatus*. In addition, MS3 and MS7 satDNA sequences were obtained from the longest autosome pair (A_1_). The reactions were performed in 1x PCR buffer, 1.5 mM MgCl_2_, 200 μM each dNTP, 0.5 U of *Taq* polymerase (Invitrogen), 0.1 μM each primer and 50 ng of DNA. The cycling program for amplification of these regions consisted of an initial denaturation at 95°C for 5 min, followed by 32 cycles at 95°C for 45 s, 56°C for 30 s and 72°C for 1 min and a final extension of 72°C for 15 min. The PCR products were visualized in 2% agarose gels, and the fragment obtained from each sample was extracted from the gel and cloned into the pGEM-T Easy Vector (Promega, Madison, Wisconsin, USA). DNA sequencing was performed with the Big Dye TM Terminator v3.1 Cycle Sequencing Ready Reaction Kit (Applied Biosystems) following the manufacturer’s instructions. Although different strategies were adopted (i.e., testing different primer pairs and adding DMSO to the PCR), we failed to amplify any region of the 45S rDNA sequence from B chromosomes, probably because GC-rich sequences are usually under-amplified in WGA steps [7].

### Extraction of MS3 and MS7 from Illumina reads

To obtain a detailed and reliable score of haplotype abundance for the MS3 and MS7 satDNAs sequences from genomic libraries, we extracted the monomers directly from the Illumina reads. Because the reads are smaller than the monomer size of both satellites, we joined the paired-reads using fastq-join (https://code.google.com/p/ea-utils/wiki/FastqJoin) with a minimum overlapping size of 6 bp.

For MS7, we aligned the joined reads against a dimer sequence of satDNA with RepeatMasker software [37] and using a custom Python script (https://github.com/fjruizruano/ngs-protocols/blob/master/rm_getseq.py); we employed the alignment information from the output file (with the extension.out) to extract only the aligned region. Then, we mapped these sequences with Geneious Pro v8.04 against the dimer and extracted the central region corresponding to one monomer by manually deleting those sequences that did not cover an entire monomer. For MS3, after Geneious mapping, we cut the ends mapped out of the reference monomer and added them to the other end to obtain sequences starting and ending at the same positions, which was achieved with another custom Python script (https://github.com/fjruizruano/ngs-protocols/blob/master/sat_cutter.py).

### Sequence analysis

Consensus sequences from forward and reverse strands were obtained using Geneious Pro v8.04, and alignments were generated using the Muscle algorithm [38] under the default parameters. DNA diversity analyses, considering indels, were performed with DnaSP v5.05 [39]. Minimum spanning trees were built on the basis of pairwise differences using ARLEQUIN v3.5.1.3 [40] and were visualized with HAPSTAR v0.7 [41].

Comparisons of synonymous substitutions per synonymous site (dS) and non-synonymous substitutions per non-synonymous site (dN) of H3.2 from each library (B_1_, B_2_, A_1_ and 0B gDNA) were carried out using the nonparametric Kruskal-Wallis test, followed by Dunn’s multiple post-hoc test, considering α = 0,05.

## Results

Individuals from the two analyzed populations showed a similar standard karyotype, all exhibiting 2n = 50 biarmed chromosomes (6m + 16sm + 28st), with no sex-related chromosomal dimorphism. Additionally, mitotically unstable B chromosomes were observed in the genomes of all studied specimens collected from the BR population, as manifested in the intraindividual variation in B numbers (0–6). However, all individuals from the NR population lacked B chromosomes.

**Fig 1:**
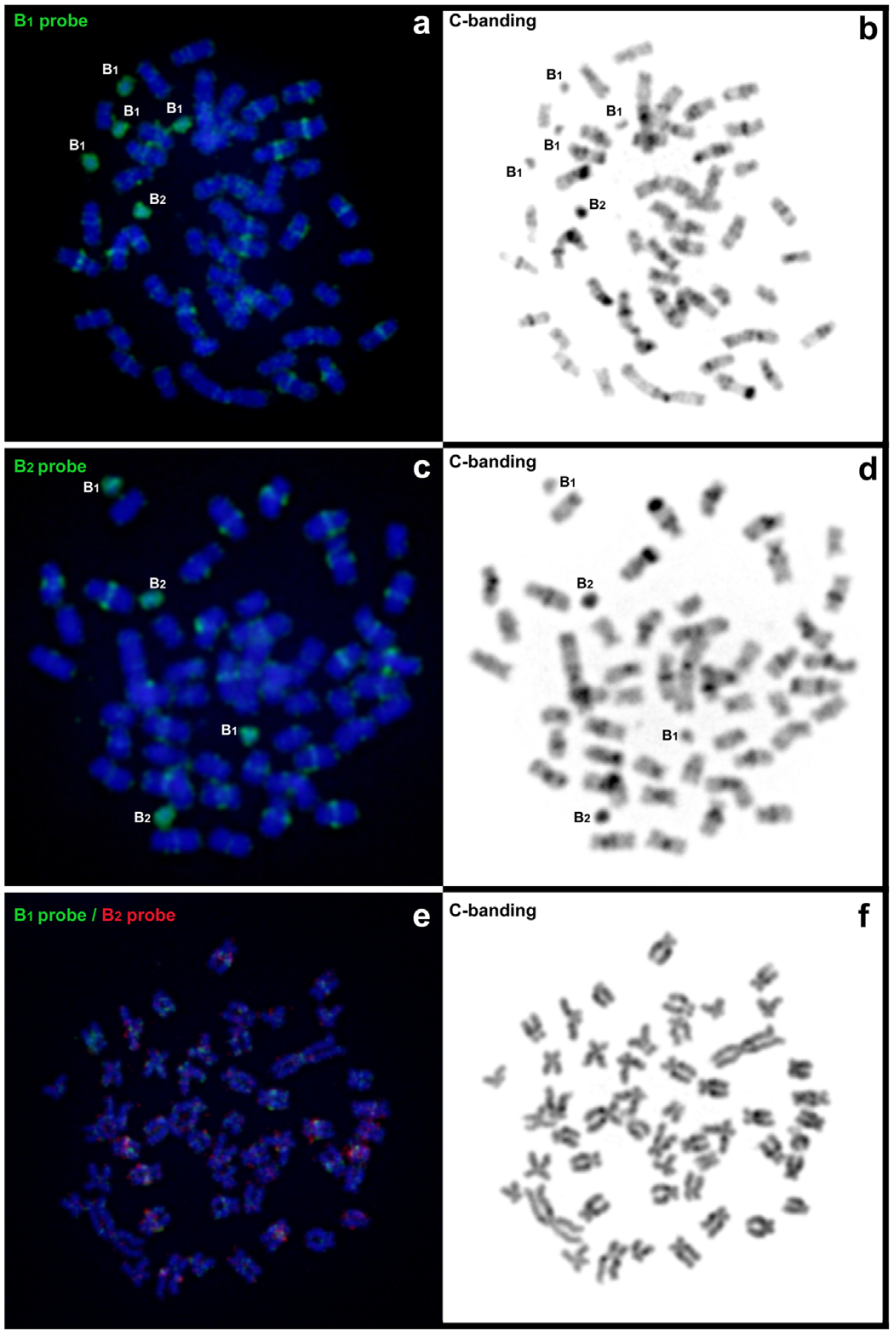
Metaphase plates of *M*. *sanctaefilomenae* after whole-chromosome painting with B_1_ and B_2_ probes and their respective sequential C-banding patterns. (a-d) represent *M*. *sanctaefilomenae* from the Batalha River. (e-f) represent *M*. *sanctaefilomenae* from the Novo River.

In the BR population, there were two types of B chromosomes observed on the basis of the C-banding pattern and population frequency. The more frequent variant (designated B_1_) showed a light C-banding pattern (similar to euchromatin in the A chromosomes), whereas the less frequent variant (B_2_) showed a dark C-banding pattern (similar to heterochromatin in the A chromosomes) (Fig 1). While B_1_ was found in all 23 individuals analyzed from the BR population (100% prevalence), B_2_ was present only in four of them (17% prevalence) (Table 1). Both B types were found in males and females.

### Similar DNA content in the two B chromosome variants

Whole-chromosome painting (WCP) with the B_1_ and B_2_ probes showed similar hybridization signals for the two probes on both B variants, in addition to the pericentromeric regions of approximately one-third of the A chromosomes (Fig 1a–1d). This was also apparent when double WCP with both B-derived probes was performed on metaphase cells from B-lacking NR individuals, with both hybridization signals co-locating in most cases (Fig 1e and 1f).

**Fig 2:**
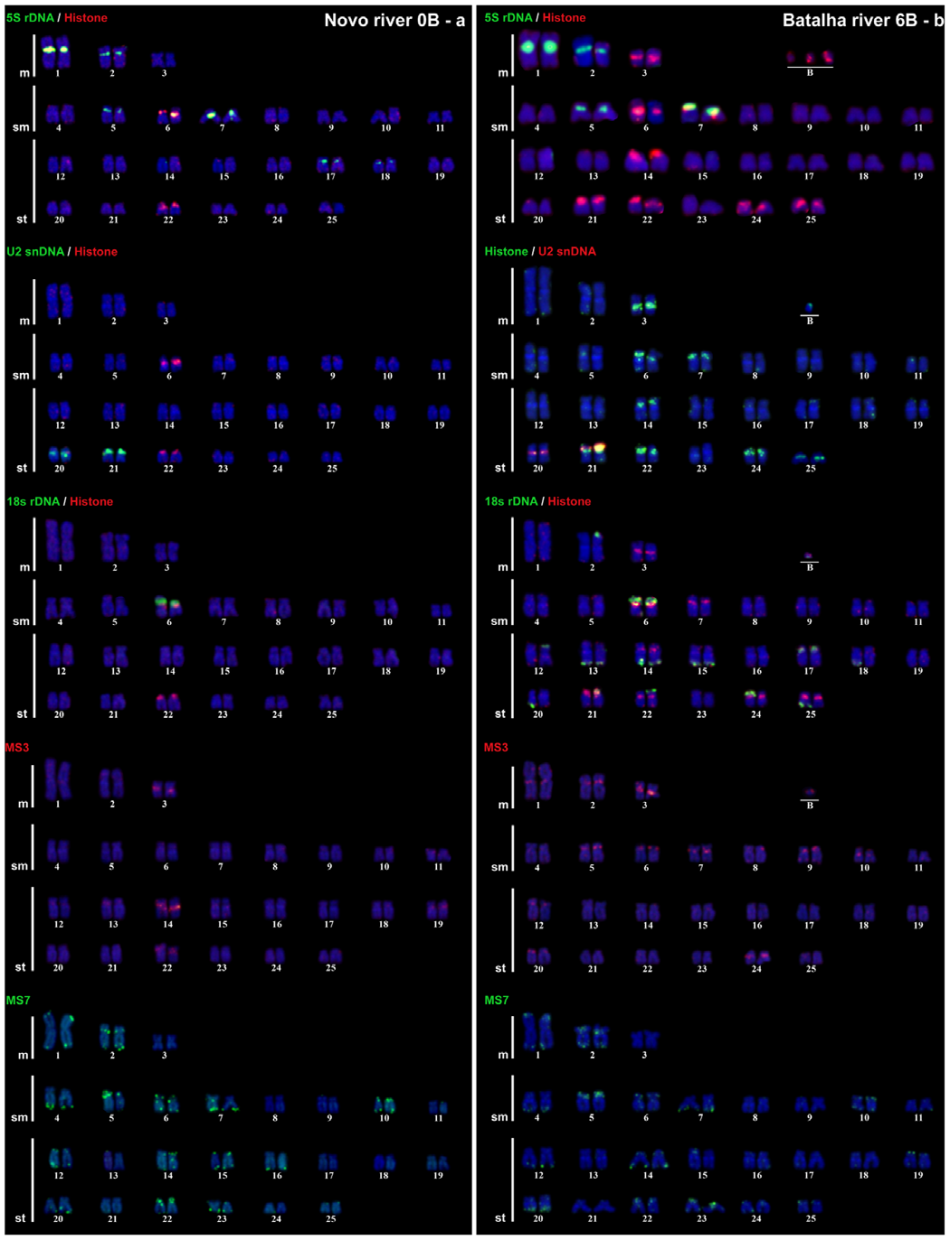
*M*. *sanctaefilomenae* karyotypes constructed from mitotic metaphase cells subjected to FISH with different repetitive DNA probes. a) Novo River population. b) Batalha River population. Note the differential distribution of H3 histone genes and 5S and 18S rDNA clusters on the A chromosomes in both populations.

FISH mapping revealed remarkable differences between the two populations regarding the number of H3 histone, 5S and 18S rDNA clusters, while the U2 snDNA showed exactly the same pattern of chromosomal distribution (Fig 2).

The most extreme difference between the two populations was found for 18S rDNA which, was located only on the short arm of chromosome pair no. 6 in NR, whereas in BR, distal clusters appeared on many chromosomes in a homozygous (13, 14, 15, 17) or heterozygous (2, 12, 20, 22, 24, 25) state, in addition to the cluster on chromosome 6. Sequential FISH and silver impregnation (with the latter indicating active rDNA clusters) showed that the rDNA cluster on chromosome 6 was always active, while most other clusters on other chromosomes (including B chromosomes) in the BR population were inactive (S1 Table, S1 Fig). Considering the B chromosomes, NOR activity was observed only for B_1_ in approximately 10% of the cells analyzed in a single individual, indicating that the contribution of the B chromosomes to rRNA synthesis does not seem to play an important role in the cellular physiology of B-carrying *M*. *sanctaefilomenae* specimens.

**Fig 3:**
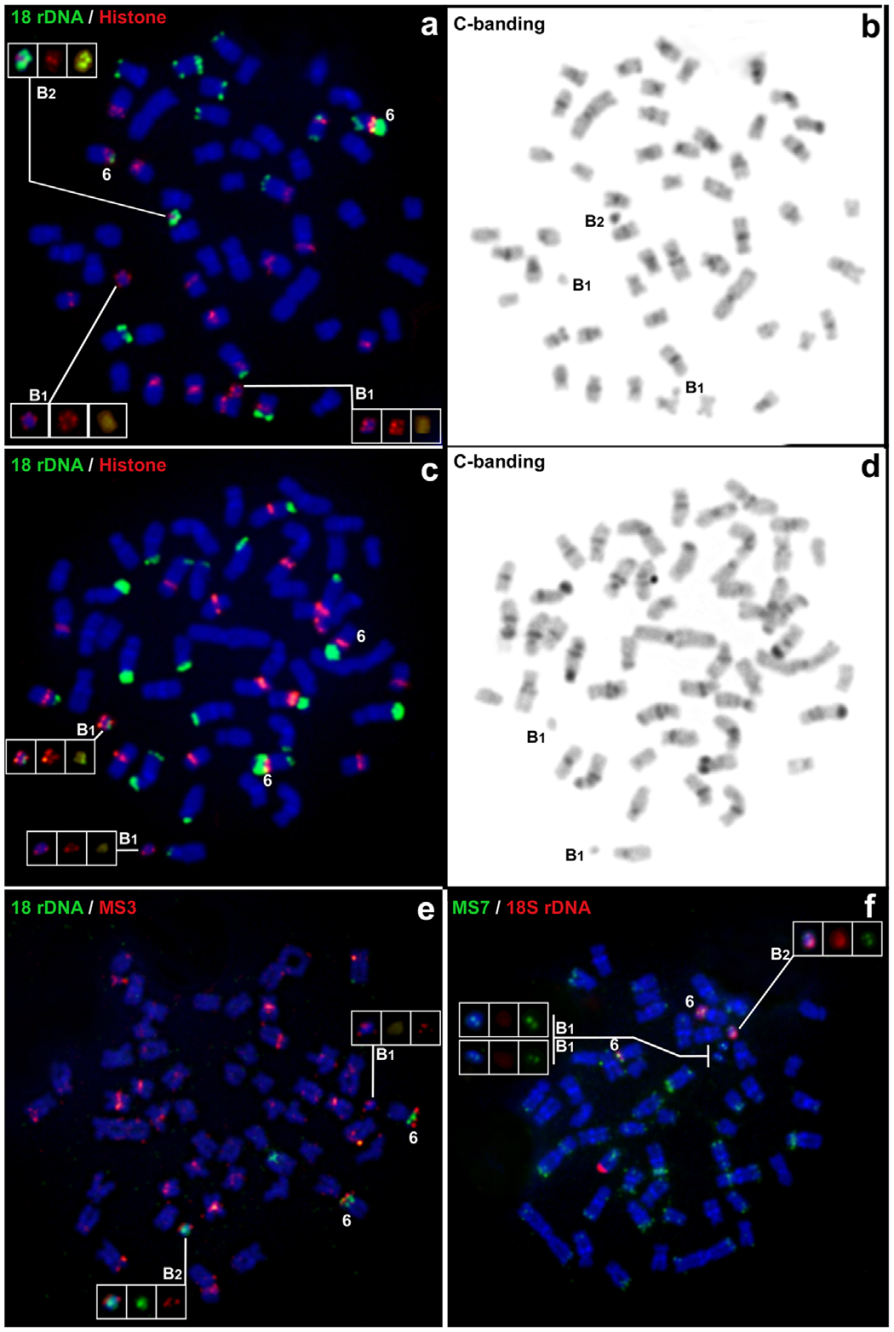
Metaphase plates of *M*. *sanctaefilomenae* from the Batalha River after FISH with different repetitive probes and sequential C-banding to show the clustering of 18S rDNA, H3 histones and satDNAs on different B chromosomes. Note that B_1_ is euchromatic showing small blocks of 18S rDNA, and B_2_ is heterochromatic showing larger amounts of 18S rDNA.

Among the tandem repeat gene families assayed, both types of B chromosomes carried only 18S rDNA and H3 histone gene sites, but the heterochromatic variant (B_2_) carried a larger 18S rDNA cluster than the euchromatic one (B_1_) (Figs 2 and 3).

To improve our knowledge of the DNA content of the B chromosomes, we searched for satellite DNA (satDNA) tandem repeats in two sets of Illumina Hiseq2000 Paired-End reads obtained from whole-*M*. *sanctaefilomenae* genome sequencing runs from a B-lacking individual from the NR population and a B-carrying individual from the BR population. Because all individuals analyzed from the BR population carried B chromosomes, it was necessary to analyse a B-lacking genome from the NR population. However, it was unfortunate that the two repeat families present on the B chromosomes (18S rDNA and H3 histone genes) showed extensive spreading across A chromosomes in the B-carrying population, thus impeding the detection of changes in DNA repeat coverage between the B-carrying and B-lacking genomes. For this reason, we used the Illumina reads to search for satellite DNAs that might be useful as additional B-specific FISH markers.

Sequence clustering analysis resulted in 11,149 clusters, constituting genomic proportions of 25.6% and 24.4% for +B and 0B individuals, respectively. We designed primer pairs for seven putative satellite DNAs, then PCR amplified these DNAs and selected those showing a ladder-like pattern in agarose gels. Next, we generated DNA probes for FISH and mapped them to A and B chromosomes (data not shown), and we selected the two satDNA families showing a clustered distribution that were present on both A and B chromosomes (Fig 3e and 3f), henceforth referred to as MS3 (CL27) and MS7 (CL96). The MS3 satDNA showed a consensus sequence of 186 bp with a cluster density of 0.16 (S2 Fig) and did not show any similarity to the custom database. Notably, this cluster was 2x more abundant in the BR library (0.236%) than in the NR library (0.117%). The MS7 satDNA exhibited a consensus sequence of 100 bp with a cluster density of 0.55 (S2 Fig) and yielded similarity hits with DNA/TcMar-Tc1 (54% of hits) in the *A*. *mexicanus* database. In terms of abundance in different libraries, there was almost no difference observed for this cluster between the two populations analyzed (0.0285% for BR and 0.0295% for NR). FISH analysis corroborated the abundance data and revealed that MS3 was located in the pericentromeric region of 13 chromosome pairs in the BR population, but only 6 pairs in the NR population. Therefore, the higher abundance of MS3 in the B-carrying population was not due to B chromosomes (even though they actually carry this satellite) but to its presence on twice as many A chromosomes. Conversely, MS7 was located in the telomeric regions of 15 pairs in both analyzed populations.

Taken together, these results indicate that both B variants contain essentially the same DNA repeats (H3 histone genes, 18S rDNA and MS3 and MS7 satDNAs). The fact that autosome pair no. 6 is the only A chromosome carrying all of these repeat families strongly points to the possibility that both B chromosomes were derived from the pericentromeric region of this chromosome.

### The two B chromosome variants show a similar degree of mitotic instability

Because the number of B chromosomes varied among cells within the same individual, we performed an analysis of the degree of mitotic instability causing this variation. For this purpose, we used a mitotic instability index previously developed in a migratory locust [42] that is based on the assumption that the median number of B chromosomes in the adult represents the number of B chromosomes in the zygote stage. This mitotic instability index (MI) measures the sum of deviations in B numbers in a sample of cells with respect to the median, normalized per B chromosome.

**Fig 4:**
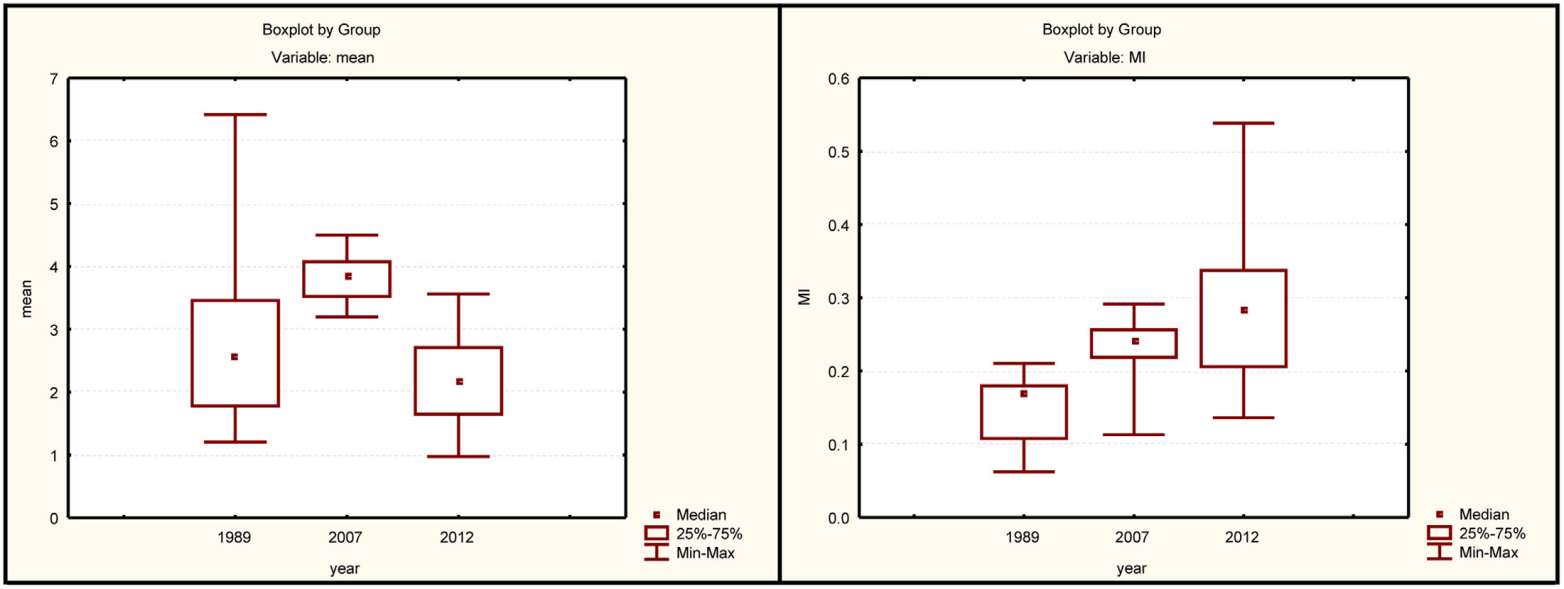
Comparison of the mean number of B chromosomes and mitotic instability index (MI) per individual between the present data and those previously reported for the Tiete River by Foresti et al. (1998) and Dantas et al. (2007).

The fact that both B chromosome types contained H3 histone genes and 18S rDNA helped us to identify the two B types in 657 mitotic metaphase cells subjected to double FISH and subsequent C-banding in 23 individuals from the BR population (mean = 29 cells per individual, SD = 6) to accurately score the number and type of B chromosomes (Fig 4). In each individual, we calculated the mean number of B chromosomes per cell, the median number of Bs and the mitotic instability index (MI). The results revealed that B_1_ and B_2_ showed a similar MI, but B_1_ was almost nine-fold more frequent than B_2_ (Table 1).

A comparison of the mean number of B chromosomes and MI per individual between the present data with those previously reported for the Tiete River in [21] and [23], by means of the Kruskal-Wallis test, revealed significant differences for both the mean values (H = 21.16, df = 2, N = 46, p< 0.0001) and MI (H = 15.4, df = 2, N = 46, P = 0.0005). Given that the three samples were collected from the same river, but at different times, these results suggest a tendency of MI to increase across years. However, we observed variation in the mean number of B chromosomes per individual that presented an inverted V-shape (Fig 4). This might suggest that the mitotic instability of B chromosomes has increased across the years, while the number of Bs per individual has reached a maximum, in accord with the existence of a tolerance threshold.

### DNA sequence analysis suggests different ages for the two B variants

Three of these repetitive sequences (H3 histone, and MS3 and MS7 satDNAs) were PCR amplified from four sources (0B gDNA from NR and microdissected B_1_, B_2_ and A_1_ chromosomes from BR). Notably, we were unable to amplify any fragment of the major ribosomal sites from the microdissected libraries or histone H3 genes from the A_1_ chromosome. These PCR products were cloned and sequenced in both directions. After discarding primer regions, a total of 318–328 bp, 183–186 bp and 93–106 bp from the H3 histone, MS3 and MS7 sequences, respectively, were obtained from several clones and Illumina reads (Table 2). To minimize the impact of possible PCR/sequencing errors, we discarded singletons from subsequent analyses. All the isolated repetitive sequences were deposited in GenBank (accession numbers as follows: MS3: KU129073-KU129201, KU177253-KU177378, MS7: KU129202- KU130117, KU177379-KU177435, Histone H3: KU184525-KU184571

**Table 2:** Genetic variation observed in MS1, MS2, H3.2 and H3.3 sequences obtained through microdissection and PCR amplification and directly from reads.

**Sequence**	**Sample**	**N**	**Length**	**Hap**	**Hd**	**π**	**Def**	
**MS3**	0B reads	129	183–184	38	0.8975	0.06334	-	
	6B reads	64	183–184	28	0.9127	0.07408	-	
	Pair No.1	19	183–185	2	0.3509	0	-	
	B_1_	15	184–185	6	0.8762	0.02174	-	
	B_2_	17	183–184	5	0.7059	0.04243	-	
	0B gDNA	6	184–186	3	0.800	0.05448	-	
	*A*. *fasciatus*	5	184	5	1	0.02391	-	
**MS7**	**Sample**	**N**	**Length**	**Hap**	**Hd**	**π**	**Def**	
	0B reads	394	96–100	110	0.9425	0.02061	-	
	6B reads	522	93–100	129	0.9266	0.02041	-	
	Pair No.1	13	98	1	0	0	-	
	B_1_	16	100	2	0.1250	0.00125	-	
	B_2_	18	100	1	0	0	-	
	0B gDNA	6	100	3	0.7333	0.01133	-	
	*A*. *fasciatus*	4	106	2	0.6667	0.00629	-	
**H3.2**	**Sample**	**N**	**Length**	**Hap**	**Hd**	**π**	**Def**	**Pol**
	B_1_	10	318–328	5	0.8222	0.01726	2	7
	B_2_	18	318–328	7	0.8627	0.01247	9	8
	0B gDNA	19	318–328	6	0.6608	0.00376	2	1
**H3.3**	**Sample**	**N**	**Length**	**Hap**	**Hd**	**π**	**Def**	
	B_1_	1	328	1	0	0	0	
	B_2_	5	328	1	0	0	0	

N = number of clones, Hap = number of haplotypes, Hd = haplotype diversity, π = nucleotide diversity per site, Def = defective copies (stop codons or gapped sequences), Pol = polymorphic sequences (sequences with different amino acids in relation to *Danio rerio* H3.2).

The H3 histone gene sequences obtained from the B types showed two distinct isotypes: H3.2 (28 clones, 11 of which showed a 10 bp deletion, and 15 were polymorphic with respect to the *Danio rerio* H3.2 amino acid sequence; UniProt accession number Q4RF4) and H3.3 (6 clones, all of which were non-defective and identical to the *Danio rerio* H3.3 amino acid sequence; UniProt accession number Q6PI20). In the case of the 0B gDNA, however, we obtained only the H3.2 isotype (19 clones, 2 of which were defective, showing a 10 bp deletion and 1 polymorphism in relation to the *Danio rerio* H3.2 amino acid sequence).

**Fig 5:**
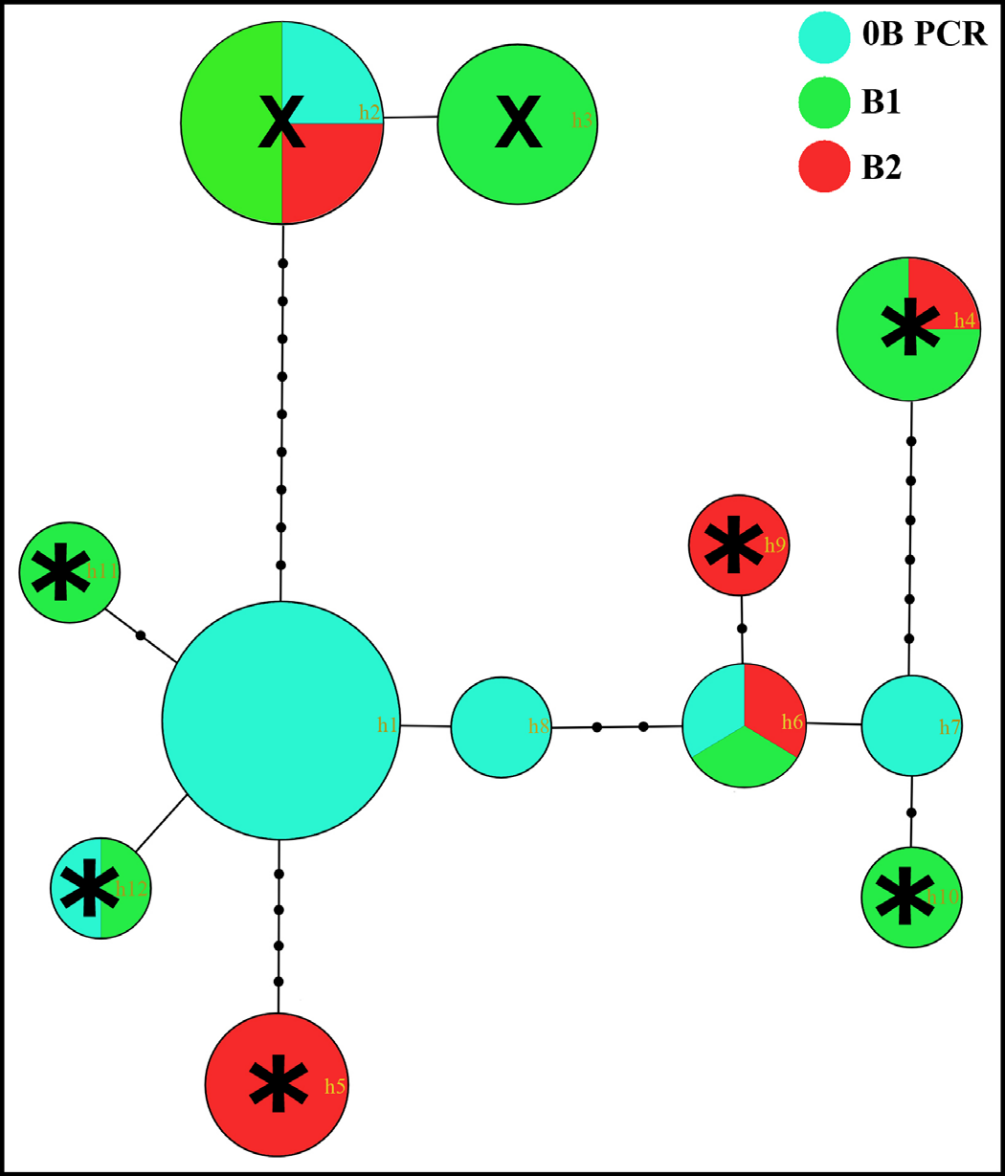
Minimum-spanning trees (MSTs) showing the relationships between the different H3.2 haplotypes obtained from different libraries. Coloured circles represent haplotypes, and each black dot represents a mutational step. “X” inside the circles indicates defective copies (a 10 bp deletion), and “*” inside the circles indicates polymorphic amino acids in comparison with the *Danio rerio* H3.2 amino acid sequence (UniProt accession number Q4RF4).

Calculation of dN for the H3.2 sequences in each group revealed significant differences between the 0B gDNA sequences and those from B_1_ and B_2_ (H = 161.7, dF = 2, N = 47, p<0.0001). Post-hoc comparisons (not shown) failed to show significant differences between the two B chromosome types, but both B_1_ and B_2_ showed significantly higher dN values compared with the H3.2 sequences from the 0B genome (Table 3). Conversely, dS showed significant differences between the 0B, B_1_ and B_2_ sequences (H = 40.12, dF = 2, N = 47, p<0.0001), and post-hoc comparisons revealed significant differences in all cases (not shown). It was remarkable that dS was almost twice as high for B_1_ than for B_2_, suggesting that the former is probably older and has had longer time to accumulate synonymous changes. In addition, the dN/dS ratios of the B_1_ and B_2_ sequences were higher than that for the 0B gDNA, suggesting that purifying selection is relaxed in the B chromosomes. The minimum spanning tree built with H3.2 haplotypes showed a certain degree of differentiation of the B-derived sequences (Fig 5).

**Table 3:** Number of synonymous and non-synonymous substitutions per synonymous (dS) and non-synonymous (dN) site, respectively, observed in the DNA sequences of H3.2 histone genes

H3.2				
Sample	N	dN	dS	dN/dS
B_1_	10	0,00623±0,004615	0,04031±0,02544	0,1545
B_2_	18	0,00626±0,00399	0,02641±0,03286	0,2370
0B gDNA	19	0,00045±0,00132	0,00699±0,00845	0,0643

**Fig 6:**
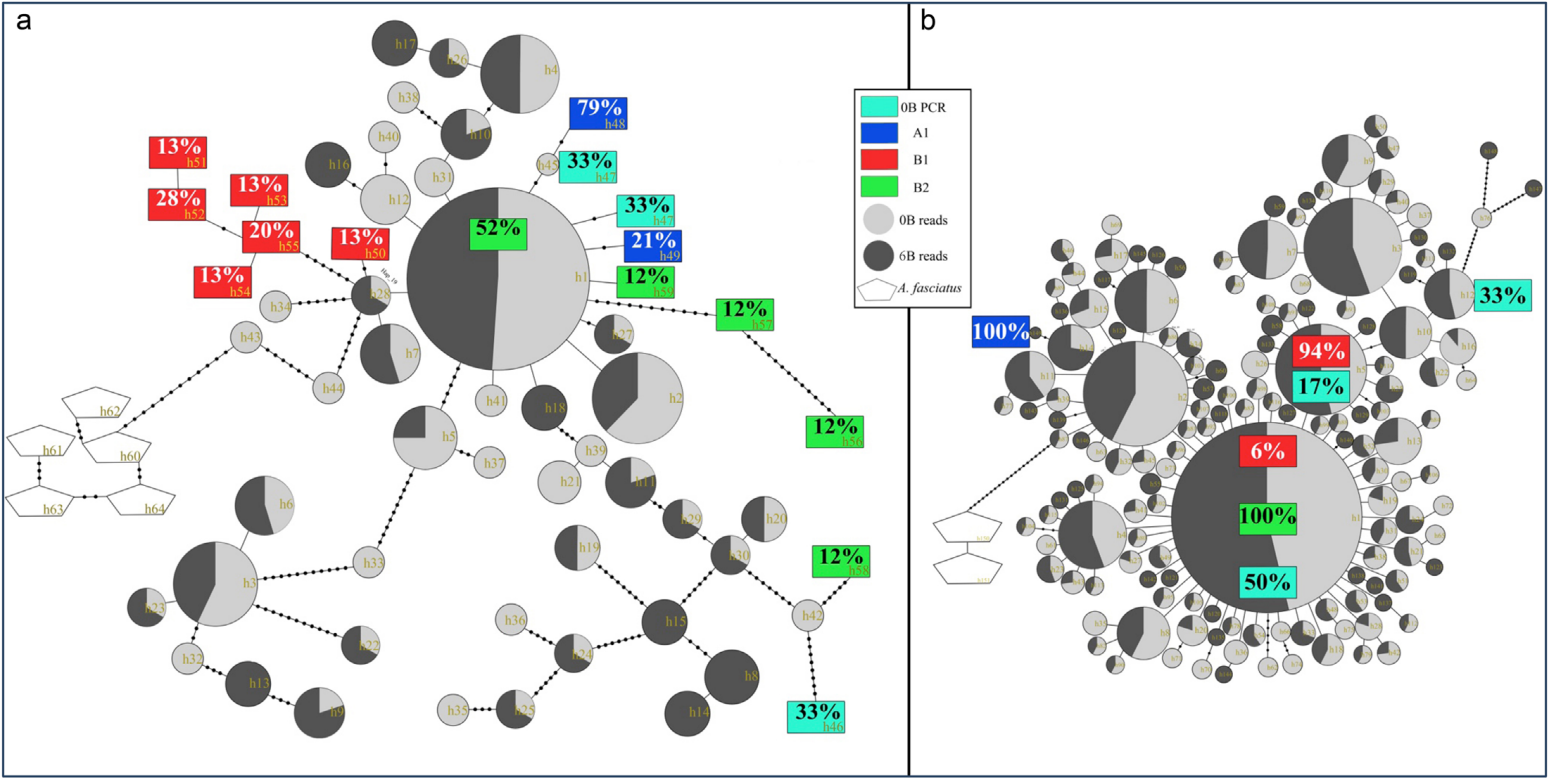
Minimum spanning trees (MST) showing the relationships between the different haplotypes of MS3 and MS7 satDNAs obtained from distinct libraries. Haplotypes retrieved directly from Illumina reads are represented by light/dark grey circles, and the diameter of the circles is proportional to their abundance, whereas PCR-amplified haplotypes are represented by coloured rectangles, with the percentage of clones corresponding to each haplotype. Each black dot represents a mutational step.

In the case of the MS3 and MS7 satDNAs, we obtained 62 and 57 PCR clones, respectively. In addition, 193 and 916 monomers were extracted from the Illumina reads for the MS3 and MS7 satDNAs, respectively (Table 2). We aligned the Illumina reads for each satDNA and built minimum spanning trees considering haplotype relative abundance, upon which we traced the PCR sequences obtained from the different libraries (Fig 6). Illumina reads are expected to provide accurate estimates of haplotype abundance, without the bias of PCR amplification. In both cases, the satDNAs amplified from *A*. *fasciatus* showed the highest divergence (as inspected in the nucleotide alignment), as expected for DNA sequences subjected to concerted evolution. The most abundant haplotypes for MS3 and MS7 in *M*. *sanctaefilomenae*, found in the Illumina reads (Fig 6), were present in the 0B and 6B genomes. However, for MS3, this haplotype was found only on the B_2_ chromosome and not on the B_1_ chromosome or in the 0B genomic DNA (PCR). The minimum spanning trees showed higher conservatism for MS7 (Fig 6b) than MS3 (Fig 6a). Remarkably, the tree for the MS3 satDNA showed that the most abundant haplotype found in the 0B and 6B Illumina-sequenced genomes was present in the PCR sequences obtained from the B_2_ chromosome, but not in those coming from the B_1_ chromosome. This difference would not be expected if one B-type was derived from the other. In addition, the absence of the most common haplotype on B_1_ and its presence on B_2_ would be consistent with an independent and more recent origin for the latter (conceivably from the same A chromosome). The tree for the MS7 satDNA was consistent with the former conclusion because all DNA sequences found on the B_2_ chromosome corresponded to the most abundant haplotype in the 0B and 6B genomes, whereas only 6% of the DNA sequences obtained from the B_1_ chromosome corresponded to the former haplotype, and the remainder belonged to a different haplotype showing one mutational difference.

## Discussion

To date, all studied populations of *M*. *sanctaefilomenae* harbour B chromosomes [21–25]. Since their first description, several authors have proposed the occurrence of three B types in this species, distinguishable by their C-banding patterns [21, 24]. Recently, it was reported that some B chromosomes in this species carry 18S rDNA clusters, while others do not [25]. In the present study, C-banding and FISH analyses allowed us to clearly identify only two different B types, which showed differences in terms of C-banding patterns, the abundance of 18S rDNA and population frequency. We also analyzed a novel population collected in the Paranapanema river basin and, for the first time in this species, no B chromosome-bearing individuals were found. In fact, B chromosomes are usually highly dynamic, and the occurrence of populations with and without B chromosomes is common. Previous studies on the grasshopper *Myrmeleotettix maculatus* have shown a cline of B chromosomes correlated with temperature or rainfall, so that supernumerary elements are absent from populations in climatically stringent environments [43, 44]. On the other hand, the geographical distribution of B chromosomes in the grasshopper *Eyprepocnemis plorans* appears to be shaped by historical non-selective events, such as the occurrence of geographical barriers that limited the spread of B-carrying individuals [45, 46]. These two B chromosome systems (in *M*. *maculatus* and *E*. *plorans*) illustrate two of the main explanations for the existence of populations lacking B chromosomes: *i*) that B chromosomes have not yet reached these populations, as in *E*. *plorans*; and *ii*) that selective constraints are acting to prevent their occurrence, as in *M*. *maculatus*. In the present study, however, the question of whether B chromosomes ever existed and were eliminated from this specific population, or B chromosomes originated after the split of these populations and have not yet reached the NR population remains open.

Individuals carrying different variants of supernumerary chromosomes have been reported in several species. Among fish, *Poecilia formosa*, *Alburnus alburnus*, *Prochilodus lineatus*, *Astyanax scabripinnis*, *A*. *goyacensis*, *M*. *sanctaefilomenae* and some cichlid fishes are the best-studied examples ([18, 21, 47–51], while the grasshopper *Eyprepocnemis plorans* constitutes the most complete known example of B chromosome diversification [52, 53]. In this last species, several studies have shown the existence of numerous B variants (more than 40) that probably arose from a common ancestral B chromosome [53, 54]. In the B chromosome system of the fish *M*. *sanctaefilomenae*, the B_1_ and B_2_ variants showed a similar degree of mitotic instability, whereas the frequency of the euchromatic variant (B_1_) was almost nine-fold higher than that of the heterochromatic variant (B_2_). Because mitotic instability is a frequent mechanism underlying drive [55], we can infer that it is not responsible for the difference between B_1_ and B_2_, although other conceivable drive mechanisms (e.g., meiotic) should be analyzed in future experiments. Nevertheless, our sequence analysis of MS3 and MS7 satellite DNAs suggests that B_1_ is older than B_2_, which would be consistent with the higher frequency of the former in the BR population. If B_2_ was actually younger, we would expect it to increase in frequency during the coming years, most likely at the expense of B_1_, because the frequency of B chromosomes appears to have reached a maximum in this population. This population therefore provides an opportunity to witness the possible replacement of one B variant for another, similar to what was previously reported in the grasshopper *Eyprepocnemis plorans* [56].

Sharing of repetitive DNAs between A and B chromosomes is a common feature, as demonstrated in various animals, including fish, grasshoppers and mammals [7, 9, 49, 57, 58]. The composition of B chromosomes has been used to identify the probable ancestral chromosome in the host species [7, 9, 35, 49]. Our WCP and FISH mapping results showed that the B_1_ and B_2_ chromosomes of *M*. *sanctaefilomenae* are composed of the same repetitive DNA sequences, suggesting a common intraspecific origin of these chromosomes. Recently, Scudeler et al. [25] suggested an intraspecific origin of B chromosomes in this species, based on WCP results indicating the presence of DNA sequences shared between A and B chromosomes. Our present results are consistent with this conclusion. However, they also suggested an independent origin for different B variants in this species because they were not painted with the only B probe employed. We cannot rule out the possibility that other B variants were present in these authors’ samples, but our present analysis revealed that all of the B chromosomes observed in the 23 individuals analyzed in the BR population contained H3 genes and 18S rDNA.

Because autosomal pair No. 6 is the only pair in the A karyotype that exhibits co-located histone, 18S rDNA, MS3 and MS7 sites (i.e., the repetitive DNA sequences contained on B chromosomes), we suggest that this pair might be the B chromosome ancestor. Remarkably, the minimum spanning trees obtained from MS3 and MS7 satDNA sequences also supported the hypothesis of an intraspecific origin due to the high similarity, and even shared haplotypes, between the sequences located on the A and B chromosomes of *M*. *sanctaefilomenae*. However, the fact that 52% of the MS3 and 100% of the MS7 sequences observed on B_2_ corresponded to the most frequent haplotype found in the Illumina reads from the B-lacking and B-carrying populations, whereas these figures were 0 and 6%, respectively, for B_1_, suggests that the two B chromosomes arose independently from autosome 6, such that in the most recent B type (B_2_), many satDNA repeats of the commonest haplotype in the A genome, found even in distant populations, are conserved. The observed differences in satellite DNA sequences between the two B variants also suggest that concerted evolution might act separately for each B type. Remarkably, MS3 and MS7 nucleotide diversity is lower in B_1_, perhaps because of its higher possibility of sequence homogenization due to its greater age and population frequency. In the grasshopper *Eyprepocnemis plorans*, males with two or more B chromosomes form chiasmated B-bivalents during meiosis [59], which helps to explain the observed variation in the amount of distally located 45S ribosomal DNA [60]. Likewise, in *M*. *sanctaefilomenae*, the frequent presence of cells with two or more B_1_ chromosomes would allow the formation of B-bivalents during meiosis and possible unequal crossovers, yielding sequence homogenization. The fact that B_1_ shows almost twice the dS value for H3.2 histone genes as B_2_ also supports the conclusion that B_1_ is older than B_2_.

Once they originate, B chromosomes are subjected to nearly the same genetic conditions that affect the molecular evolution of sex chromosomes, particularly regarding the degeneration of the heteromorphic Y or W chromosomes, which includes loss of both functional loci and sequence homology with the regular genome as well as heterochromatin gains [3, 61]. In this context, if B_2_ was derived from B_1_, it would be unexpected for the younger B chromosome (B_2_) to be heterochromatic. However, their divergent C-banding responses might not be associated with their relative ages, but with their independent origins and differences in 18S rDNA contents. In this context, the heterochromatic nature of B_2_ seems to be related to the large amounts of rDNA-associated heterochromatin on this chromosome.

B chromosomes containing 18S rRNA genes have been described in different fish species [9, 62], but their activity has only been demonstrated for the euchromatic variant (B_1_) in *M*. *sanctaefilomenae* [24]. Several other examples of NOR-bearing B chromosomes have been described in different organisms reviewed in [61], and there is no general trend between the C-banding response and NOR activity of the B chromosomes. For example, the variants B_1_ and B_24_ in the grasshopper *E*. *plorans* are C-band positive, show active NORs and exhibit no correlation between rDNA content and NOR activity [60, 63–66], whereas in the plants *Allium flavum* and *Crepis capillaris*, the active NOR sites are located outside the constitutive heterochromatin on the B chromosomes [67, 68]. It is noteworthy that the main structural difference between the eu- (B_1_) and heterochromatic (B_2_) variants is the higher content of rDNA in the latter. Similarly, in the A chromosomes of different salmonid species, rDNA loci with smaller FISH signals show faint C-band heterochromatin, while larger clusters are coincident with strongly positive C bands [69, 70]. This condition is probably related to the interspersed organization of the rDNA within the repeated DNA sequences of the heterochromatin, although the possibility that the rDNA itself contributes to heterochromatin formation cannot be discarded [69]. In this context, the B chromosomes of *M*. *sanctaefilomenae* therefore provide a model to test the relationship between rDNA and heterochromatin contents and their roles in determining the C-banding pattern.

Other multigene families have also been found on the B chromosomes of several species [7, 9, 71]. Regarding histone genes, only the H3.2 subtype has hitherto been reported on B chromosomes [7, 9]. In *M*. *sanctaefilomenae*, two non-defective (thus, potentially active) H3 histone types (H3.2 and H3.3) were found on the eu- and hetero-chromatic B chromosome variants. Remarkably, while H3.2 was represented by several haplotypes on both B-types, the six H3.3 clones analyzed from the two B chromosome variants showed exactly the same DNA sequence. Interestingly, the coding nature of histone genes allows additional inferences to be made about the DNA sequences contained in the B chromosomes in terms of the dN/dS ratio. In general, higher dN/dS ratios are expected for coding DNA sequences residing on B chromosomes compared with the same sequences on the A chromosomes because selection is assumed to be relaxed in B chromosomes due to their dispensable nature. This situation has been reported for the H3 and H4 histone genes of the grasshopper *L*. *migratoria*, where the dN/dS ratios are 2,23 and 1,72 higher for the B chromosome, respectively [7], and for the H1 histone gene of the fish *A*. *paranae*, where the dN/dS ratio is 3 times higher for the B chromosome [9]. Our present results are consistent with these previous observations because they showed 5.77 (B_1_) and 3.78 (B_2_) higher dN/dS ratios for the B chromosomes compared with the 0B genome, indicating that purifying selection is relaxed for the H3.2 genes located on the B chromosomes. This suggests that the H3.2 genes on the B chromosomes are most likely inactive. Conversely, H3.3 genes appear to be conserved on B chromosomes. It is conceivable that the complete identity of the six DNA sequences analyzed could be due to purifying selection, thus implying the possible functionality of the B chromosome copies, but further research will be necessary to address this issue.

In conclusion, analysis of the B chromosome contents of several types of repetitive DNA sequences (18S rDNA, H3 histone genes and two satellite DNAs) and comparison with those of the A chromosomes by means of FISH mapping, chromosome painting, and DNA sequencing (by Sanger and Illumina methods) revealed that the Neotropical fish *M*. *sanctafilomenae* harbours two B chromosome variants differing in their C-banding patterns, frequency and abundance of 18S rDNA. Both B variants were presumably derived independently from the same A chromosome (autosome no. 6), but the heterochromatic variant shows signs of being younger than the euchromatic variant. Finally, both B variants showed higher dN/dS ratios for the H3.2 histone gene, suggesting that purifying selection is relaxed for the B-sequences, as expected if they are mostly inactive.

## Supporting Information

S1 Fig Metaphase plates of *Moenkhausia sanctaefilomenae* after FISH with 18S rDNA and H3 histone genes probes and sequential Ag-NOR staining. Note the differential distribution of NOR sites within the same population.
(TIF)

S2 Fig Cluster-graphs obtained for the MS3 and MS7 satDNAs after analysis with RepeatExplorer. (TIF)

S1 Table Intrapopulational polymorphism of 18S rDNA location in *M*. *sanctaefilomenae*. Gold shaded, the constant Ag-NOR pair No. 6.
(DOCX)
