# Supplementary figures and images for: Uncovering the Ancestry of B Chromosomes in Moenkhausia sanctaefilomenae (Teleostei, Characidae)
Source: PLoS One. 2016 Mar 2;11(3):e0150573. doi: 10.1371/journal.pone.0150573 (PMC4775049; doi:10.1371/journal.pone.0150573)

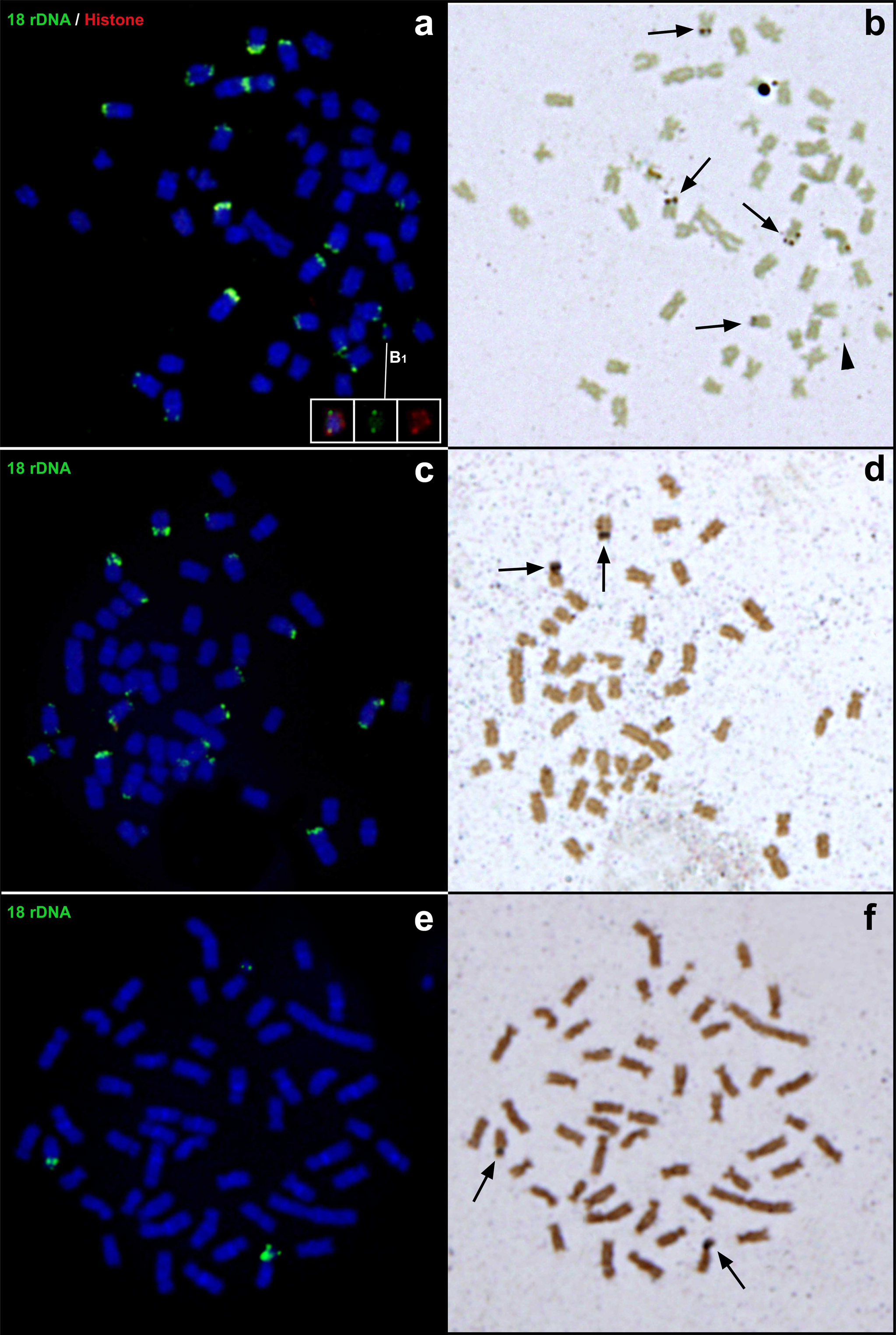

Supplement: S1 Fig — Note the differential distribution of NOR sites within the same population. (TIF) [file pone.0150573.s001.tif]

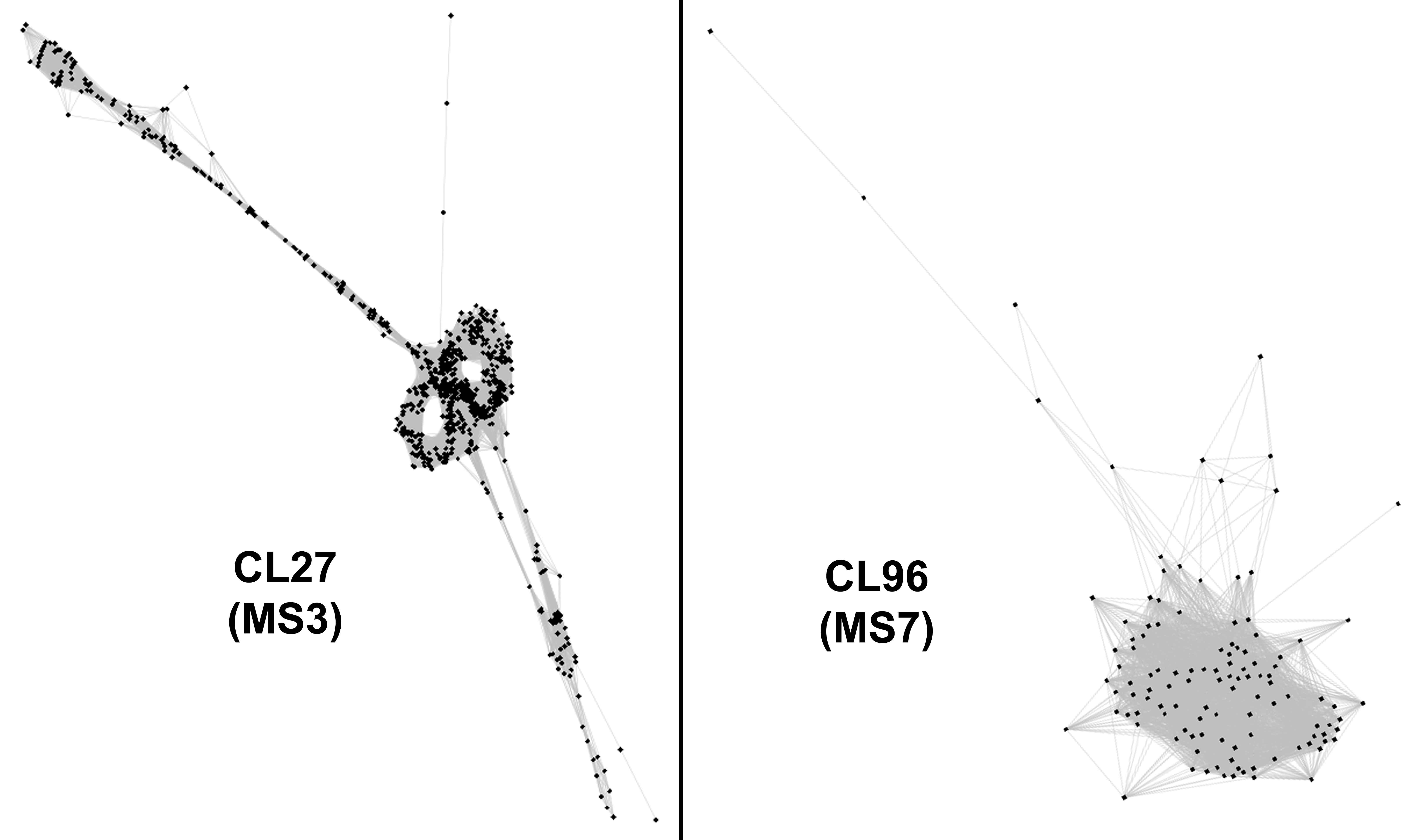

Supplement: S2 Fig — (TIF) [file pone.0150573.s002.tif]
