# Supplementary material for: Uncovering the Ancestry of B Chromosomes in Moenkhausia sanctaefilomenae (Teleostei, Characidae)
Source: PLoS One. 2016 Mar 2;11(3):e0150573. doi: 10.1371/journal.pone.0150573 (PMC4775049; doi:10.1371/journal.pone.0150573)
Supplement: S1 Table — Gold shaded, the constant Ag-NOR pair No. 6. (DOCX) [file pone.0150573.s003.docx]

Table I: Intrapopulational polymorphism of 18S rDNA location in *M*. *sanctaefilomenae*. Gold shaded, the constant Ag-NOR pair No. 6.

| Samples | Chromosomes | | | | | | | | | | | | | | | | | | | | | | | | |  |
| --- | --- | --- | --- | --- | --- | --- | --- | --- | --- | --- | --- | --- | --- | --- | --- | --- | --- | --- | --- | --- | --- | --- | --- | --- | --- | --- |
|  | 1 | 2 | 3 | 4 | 5 | 6 | 7 | 8 | 9 | 10 | 11 | 12 | 13 | 14 | 15 | 16 | 17 | 18 | 19 | 20 | 21 | 22 | 23 | 24 | 25 | Total |
| 69615 | - | - | - | p* | q | p | q* | p | - | - | p* | pq* | q* | - | q* | - | - | - | - | - | - | p | - | - | - | **14** |
| 69616 | - | - | - | - | - | p | - | q* | - | - | - | q* | - | - | q* | p* | p | - | - | - | q* | - | - | p* | q* | **11** |
| 69618 | - | - | - | q* | - | p | - | - | - | - | - | - | q* | - | - | - | - | - | - | - | - | - | - | - | - | **4** |
| 69626 | - | p* | - | - | - | p | - | - | - | - | - | p* | q* | - | q* | q* | - | - | - | p* | - | - | q* | - | - | **9** |
| 69628 | - | - | q* | - | - | p | - | - | - | - | - | q | - | - | q* | p | - | - | - | - | - | p* | - | - | - | **9** |
| 69676 | - | - | q* | - | q* | p | - | - | - | - | - | q* | q* | - | q | - | - | - | - | - | - | - | - | - | - | **8** |
| 69678 | - | - | - | - | - | p | - | - | - | - | - | - | q* | - | - | - | - | - | - | - | - | - | - | - | - | **3** |
| 69679 | - | pq* | q* | - | - | p | - | - | - | - | - | pq* | q* | - | q* | - | - | q* | - | - | - | - | - | - | - | **8** |
| 69680 | - | - | - | - | - | p | - | - | - | - | - | - | - | - | - | - | - | - | - | - | - | - | - | - | - | **2** |
| 69682 | - | - | - | q* | - | p | - | - | - | - | - | - | q* | - | q | - | - | - | - | - | - | - | - | - | - | **6** |
| 69636 | - | pq* | - | q | pq | p | - | - | - | - | - | - | q* | p* | - | pq* | - | q* | - | p* | - | p* | - | - | - | **13** |
| 69637 | - | - | - | q | - | p | - | q* | - | - | - | - | - | - | - | - | - | - | - | - | - | - | - | - | - | **5** |
| 69693 | - | - | q* | - | - | p | - | - | - | - | - | - | - | - | - | - | - | - | - | - | - | - | - | - | - | **3** |
| 69694 | - | - | q* | - | - | pq | - | p* | - | - | p | - | q* | - | q* | - | - | - | - | - | - | - | - | - | - | **8** |
| 69695 | - | - | - | - | - | p | - | - | - | - | - | - | q* | - | - | - | - | - | - | - | - | - | - | - | - | **3** |
| 69696 | - | - | - | - | - | p | - | - | - | - | - | - | - | - | - | - | - | - | - | - | - | - |  |  |  | **2** |
| 69710 | q* | p* | - | pq* | - | p | - | q* | - | - | - | - | - | - | - | - | - | - | - | - | - | - | - | - | - | **6** |
| 69711 | - | p* | - | q* | - | p | - | q* | - | - | - | q* | q* | - | - | - | - | q | - | - | - | - | - | - | - | **9** |
| 69712 | - | - | - | q* | - | p | - | - | - | - | - | pq* | - | p* | - | - | - | - | - | - | - | - | - | - | - | **5** |
| 69713 | - | - | - | - | p | p | - | - | - | - | - | - | q* | p* | - | - | - | - | - | - | - | - | - | - | - | **6** |
| 69714 | - | - | q* | - | - | p | - | - | - | - | - | p* | q* | - | - | q* | - | - | - | - | - | - | - | - | - | **6** |
| 69729 | - | - | - | - | p* | p | - | - | - | - | - | - | q | - | - | q* | p* | - | - | - | - | - | - | - | - | **7** |
| 69730 | - | - | - | q* | - | p | - | - | - | - | - | pq* | - | p* | - | - | - | - | - | - | - | - | - | - | - | **5** |
| 69731 | - | p* | - | - | - | p | - | - | - | - | - | p* | q | q | q | q | p | q* | - | q* | - | p* | - | p* | q* | **19** |
| 69732 | - | - | q* | - | - | p | - | - | - | - | - | q* | - | p* | q* | p* | - | - | - | p* | - | - | - | - | - | **8** |
| 69739 | - | - | q* | p* | - | pq* | - | - | - | - | - | p* | - | pq | - | p* | - | - | - | - | - | - | - | p | - | **10** |
| 69740 | - | q* | q* | pq* | - | pq* | pq* | - | - | - | - | q | q | p* | - | p* | - | - | - | - | - | - | - | - | - | **12** |
| 69741 | - | - | - | - | q* | p | - | - | - | - | - | p | - | - | - | - | p* | - | - | - | - | - | - | - | - | **6** |
| 69743 | - | - | - | q* | - | pq* | q* | - | - | - | - | - | - | p | - | p* | - | - | - | - | - | - | - | - | - | **7** |

p: short arms, q: long arms, pq: both arms, *: heteromorphic pair
